# Short Interrupted Repeat Cassette (SIRC)—Novel Type of Repetitive DNA Element Found in *Arabidopsis thaliana*

**DOI:** 10.3390/ijms241311116

**Published:** 2023-07-05

**Authors:** Igor V. Gorbenko, Ivan S. Petrushin, Andrey B. Shcherban, Yuriy L. Orlov, Yuri M. Konstantinov

**Affiliations:** 1 Cell Biology and Bioengineering, Siberian Institute of Plant Physiology and Biochemistry SB RAS, Irkutsk 664033, Russia; gorbenko@sifibr.irk.ru (I.V.G.); ivan.kiel@gmail.com (I.S.P.); yukon@sifibr.irk.ru (Y.M.K.); 2 Department of Business Communications and Informatics, Irkutsk State University, Irkutsk 664033, Russia; 3 Institute of Cytology and Genetics SB RAS, Novosibirsk 630090, Russia; atos@bionet.nsc.ru; 4 Kurchatov Genomic Center ICG SB RAS, Novosibirsk 630090, Russia; 5 The Digital Health Institute, I.M. Sechenov First Moscow State Medical University of the Ministry of Health of the Russian Federation (Sechenov University), Moscow 119991, Russia; 6 Agrarian and Technological Institute, Peoples’ Friendship University of Russia, Moscow 117198, Russia; 7 Biosoil Department, Irkutsk State University, Irkutsk 664003, Russia

**Keywords:** repetitive DNA, mobile genetic elements, short-interrupted repeats cassette, miniature inverted repeats transposable element, *Arabidopsis thaliana*

## Abstract

Short interrupted repeat cassette (SIRC)—a novel DNA element found throughout the *A. thaliana* nuclear genome. SIRCs are represented by short direct repeats interrupted by diverse DNA sequences. The maxima of SIRC’s distribution are located within pericentromeric regions. We suggest that originally SIRC was a special case of the complex internal structure of the miniature inverted repeat transposable element (MITE), and further MITE amplification, transposition, and loss of terminal inverted repeats gave rise to SIRC as an independent DNA element. SIRC sites were significantly enriched with several histone modifications associated with constitutive heterochromatin and mobile genetic elements. The majority of DNA-binding proteins, strongly associated with SIRC, are related to histone modifications for transcription repression. A part of SIRC was found to overlap highly inducible protein-coding genes, suggesting a possible regulatory role for these elements, yet their definitive functions need further investigation.

## 1. Introduction

Repetitive DNA sequences comprise the majority of plant genomes, up to 90–95% of the nuclear DNA [1,2]. Repetitive sequences are highly heterogeneous and include thousands to tens of thousands of families, which vary in motif length, copy number, and arrangement in the genome [3,4,5,6]. The causes of the maintenance of these huge amounts of repetitive DNA and their broad diversity are still poorly understood. The predominant part of the plant genome consists of interspersed repetitive DNA sequences. Most of them are formed as a result of the activity of mobile genetic elements (MGEs) belonging to two classes. Elements of class I, or retrotransposons, migrate by a “copy-and-paste” mechanism via RNA intermediates. Elements of class II are DNA transposons, which utilize a “cut-and-paste” mechanism [7].

Retrotransposons are subdivided into LTR and non-LTR retrotransposons, depending on the presence or absence of long terminal repeats (LTRs). The inner region of retrotransposons usually has two open reading frames (ORFs) coding the proteins of virus-like particles, reverse transcriptase (RT), integrase (INT), and other proteins. In plants, LTR retrotransposons are the predominant group of MGEs. They constitute from 15% (*A. thaliana*) to 90% (some Liliaceae species) of the genome [8,9,10]. Plants with large genomes (e.g., maize, wheat) may have thousands of LTR retrotransposon families. However, the majority of interspersed DNA repeats in a particular genome generally belong to a few or even one retrotransposon family, for example, *BARE1* in barley [11] or *Opie* in maize [12]. 

DNA transposons are generally less abundant, but some of them have propagated more successfully, e.g., *CACTA* in wheat [13]. This class of MGEs is subdivided into two subclasses. Subclass I includes classical MGEs having terminal inverted repeats (TIRs) of various lengths. Their transposition is affected by the transposase enzyme encoded by the autonomous elements, which recognizes TIRs and cleaves both strands at both ends of the element. Subclass II migrates by means of a rolling circle mechanism [14]. MGEs of subclass II in plants are represented by elements of the *Helitron* superfamily, which have been comprehensively described in the maize genome [15]. The ends of these elements have no TIRs but have TC or CTRR motifs (where R = purine). The autonomous *Helitron* elements encode a tyrosine recombinase of the Y2 type with a helicase domain, able to initiate replication [15]. It is worth mentioning that many *Helitron* elements occasionally capture fragments of host genes. Some groups of DNA transposons remain unclassified because the sequences of only their nonautonomous variants are known. 

It is well known that MGEs play an important role in genome evolution and genomic adaptation processes [16,17,18,19,20,21,22]. They react to many environmental or internal genotypic factors by changing their transpositional activity, which leads to various genome reorganizations both at the gene and chromosome levels [23,24,25,26]. Traces of MGE insertions have been found in the regulatory and coding regions of most of the known plant genes [27]. In allopolyploid genomes, such insertions can lead to structural divergence of homeologous genes towards sub- or neofunctionalization [28]. The mechanisms of this divergence are different: from changes in protein structure or modification of regulatory sites that control gene expression [29] to epigenetic changes in chromatin in the insertion region [25,30]. A very interesting feature of MGEs is that they can encode small RNAs that can influence the expression of individual genes [31], guide DNA methylation, and modify histones [32]. Moreover, species-specific MGEs are nowadays considered as higher-order control elements that govern ncRNA expression patterns [33]. However, it should be noted that the obvious insertions of certain MGEs that occurred relatively recently make up only a small part of the genome, while in the bulk, traces of ancient insertions are difficult to detect and analyze due to the past long-term divergence.

In the post-genomics era, with the introduction of next-generation sequencing technology, it is possible to make precise assemblies of repetitive regions of complex eukaryotic genomes and to decipher the functional potential of these regions. Whole genome sequencing showed that the complexity of the repeatome can be highly variable between plants, and therefore different species represent different challenges in terms of the search and annotation of various repeats of genomic DNA. It is known that in the background of high transposon activity, there is an increased level of interelement homologous recombination, leading to the loss of the intervening DNA. As a result, a major part of the intergenic material that contains older repeats can be deleted from the genome [34]. A model organism such as *Arabidopsis thaliana* is eligible for the search and analysis of repetitive DNA of different ages and origins because its genome is small and shows low recent MGE activity [35,36]. 

During the analysis of miRNA ath-MIRf10275 primary transcript, obtained from PMRD (plant microRNA database [37] at http://bioinformatics.cau.edu.cn/PMRD/ (accessed on 13 June 2023)), we found that the template for primary transcript contains four imperfect direct repeats (that includes mature miRNA; the scheme is shown in Supplementary Figure S1) interspersed with DNA sequences that have no similarity between each other. We designated such a structure as a Short Interrupted Repeats Cassette (SIRC). Using special software, we detected more than three thousand similar structures in the nuclear genome of *A. thaliana*. The genome distribution of SIRCs indicates a possible involvement of MGEs in their origin. The objectives of this study were to analyze the structural polymorphism and chromosome location of SIRCs, their overlapping with different genomic annotations including MGEs, genes, small noncoding RNA, and so on, and their association with DNA-binding proteins taking part in epigenetic maintenance of the genome. Resolving all these tasks will allow us to understand the functional role and properties of SIRCs, as well as the evolutionary history of this repetitive DNA element.

## 2. Results

### 2.1. The Basic Properties of SIRCs

**Figure 1:**
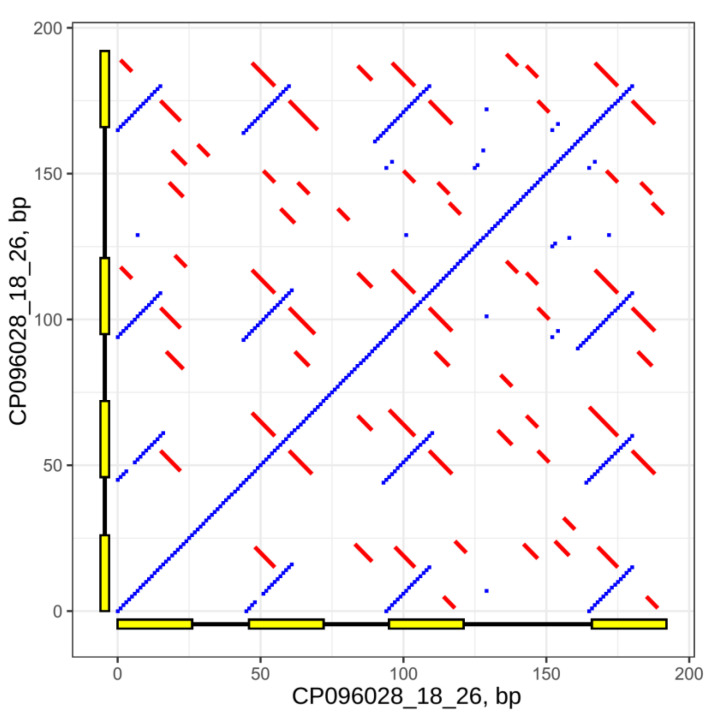
Self-similarity dotplot for SIRC CP096028_18_26, window size = 10, match bp = 8, min palindrome arm length = 5, yellow rectangles are SIRC direct repeats. Blue dots are regions of 10 bp that have a minimum of 8 bp that are identical. Red lines are palindromes with a minimal arm length of 5 bp. The rulers show sequence lengths in bp.

We detected 3050 sequences composed of 2–8 direct repeats interspaced with diverse DNA in the nuclear genome of *A. thaliana* Col-0 (Col-CEN assembly), which we named SIRCs. It should be especially noted that SIRCs are undetectable using standard repeat-detection software (e.g., RepeatMasker or the DECIPHER function detectRepeats). The dotplot of one of the SIRCs, possessing direct repeats of 26 bp length, is presented in Figure 1.

The total length of the SIRCs is 265,855 bp and comprises 0.2% of the Col-CEN genome size. The repeats of detected SIRCs were 10–29 bp, with a maximum distribution of 13 bp. SIRCs quantity per 1 Mbp was found to be significantly different in chromosomes 1, 2, and 4 vs. chromosome 5 (Wilcoxon test with Bonferroni correction, *p* = 0.0027, 0.031, and 0.0001 relatively) and in chromosome 3 vs. chromosome 4 (*p* = 0.017). The median SIRC numbers per 1 Mbp for chromosomes 1–5 are 21, 20, 14, 27.5, and 13. Statistics of DR lengths and GC-content are presented in Supplementary Figure S2 and are listed in Supplementary Data S3. The direct repeats of 1666 SIRCs (approximately 57%) do not possess palindromes; 1064 contain palindromes with arm lengths of 3 bp and 211 with arm lengths of 4–9 bp (Supplementary Figure S4).

### 2.2. Genomic Location

**Figure 2:**
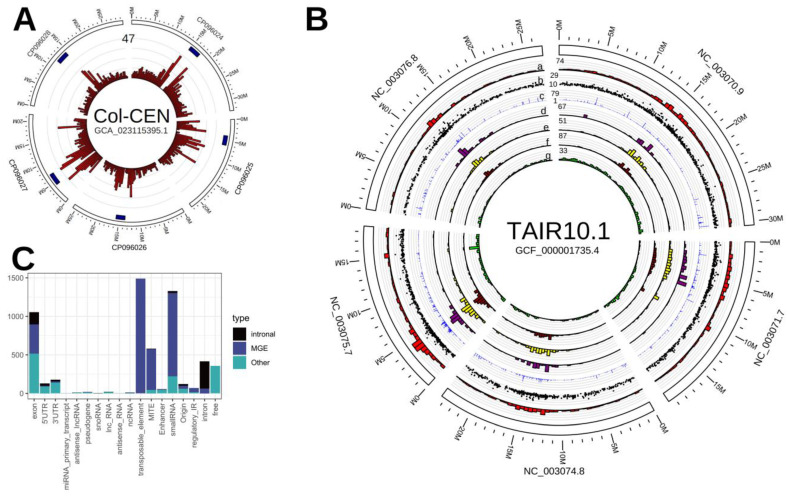
(**A**) Circos-plot of 3050 SIRCs detected in Col-CEN assembly, centromeres are marked as blue rectangles, red histogram represents the density of SIRCs per 0.5 Mbp of a genome, 47 is the maximal value of density; (**B**) circos-plot of 2941 SIRCs remapped to TAIR10.1: a—SIRCs density per 0.5 Mbp; b—SIRCs direct repeat lengths; c—SIRCs in-genome copy number (with 5 mismatches allowed); d—SIRCs overlapping MITEs per 0.5 Mbp; e—SIRCs overlapping small RNAs per 0.5 Mbp; f—SIRCs overlapping MGEs per 0.5 Mbp; g—SIRCs overlapping genes (Araport11) per 0.5 Mbp; (**C**): the number of SIRCs overlapping genomic annotations.

It is clear that the maximum number of SIRCs are located in the pericentromeric regions, while centromeres themselves possess no or few SIRCs (Figure 2A). Acrocentric chromosomes (II and IV) have additional maxima of SIRC distribution beyond pericentromeric regions—their positions are near 13.5 Mbp and 18.5 Mbp, respectively. According to different studies [38,39], the Arabidopsis ancestor had more chromosomes, and several chromosome fusion events occurred in the species’ history. Therefore, it is possible that these additional maxima of chromosomes II and IV represent areas that were pericentromeric in ancient separate chromosomes millions of years ago. It was found that SIRCs with longer palindrome arms are predominantly located in pericentromeric regions. However, nonpalindromic and SIRCs with short palindromes have similar distributions across chromosomes (Supplementary Figure S4).

The SIRC dataset was remapped from Col-CEN to the reference genome TAIR10.1 since it has a comprehensive annotation. The number of remapped SIRCs was 2941 (Figure 2B,C, the coordinates and features are presented in Supplementary Data S5).

The highest number of SIRCs overlap with mobile genetic elements (Figure 2C, the full data on overlaps of SIRCs with any annotations is presented in Supplementary Data S6). The primary transcripts of small RNAs contain more than 1200 SIRCs. Further investigations showed that most of these small RNAs are MGE-derived and are heterochromatic siRNAs (hc-siRNAs) that are utilized for transcriptional gene silencing (TGS) of repetitive regions and R-genes [40]. Therefore, SIRC fragments are apparently present in hc-siRNAs.

**Figure 3:**
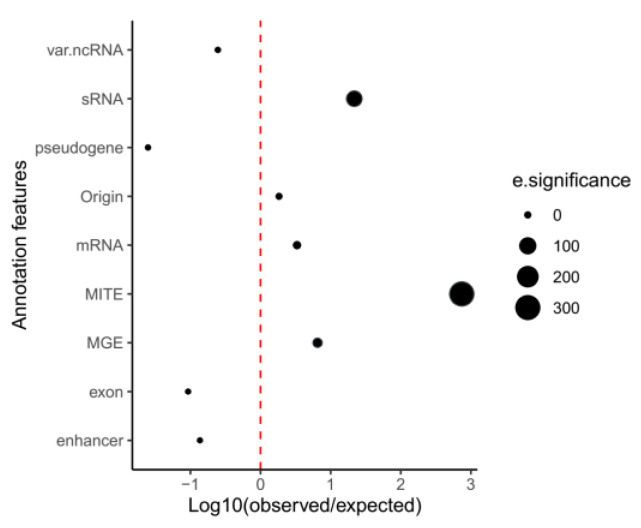
Positional enrichment analysis of SIRCs with genomic annotations. Var.ncRNA = miRNA, miRNA_primary_transcript, tRNA, antisense_lncRNA, snRNA, ncRNA, rRNA; Pseudogene = pseudogene, pseudogenic_exon, pseudogenic_transcript, pseudogenic_tRNA; MGE = transposable_element, transposable_element_gene, transposon_fragment.

We estimated positional enrichment of SIRCs that overlaps with different genomic annotations (the enrichment score is Log_10_ (observed/expected) in Figure 3). SIRCs are significantly enriched only with MGEs and small RNAs (most of which are expressed by MGEs), and the highest enrichment was found for MITEs. However, enrichment with mRNA is affected by the fact that MGE-related genes are also annotated as mRNAs, which is why we do not consider that enrichment significant. On the other hand—apparently exons and various noncoding RNAs contain fewer SIRCs than expected—perhaps those SIRCs are not preferentially inserted in these regions. The lowest enrichment score (negative) was found for pseudogenes—indicating that SIRCs are not part of some pseudogenization mechanism. The full data on positional enrichment of SIRC with genomic annotations is presented in Supplementary Data S7.

To test if the *Arabidopsis thaliana* genome possesses any sequences similar to SIRC but undetectable due to repeat mismatches, we estimated the arbitrary copy numbers of SIRC sequences with a tolerance of five mismatches (considering the full sequence, the number of mismatches was defined randomly)—it was found that some SIRCs have “hidden” copies that were altered to, such an extent that they become unrecognizable as SIRC. Of 2941 SIRCs, 26% have hidden copies, 10% have more than 5 copies, and 4%—more than 15 copies. The total number of hidden copies was 2551. Hidden copies are located mostly in MGEs: 1236 in MITEs, 683 in LTR/Gypsy (*ATHILA*, *ATGPN*, *ATLANTYS*), 298 in DNA/MuDR (*ARNOLD*, *VANDAL*, *MU*), and 286 in RC/Helitron (*ATREP*, *HELITRON*). Full statistics of detected hidden copy occurrence in non-MITE MGEs are presented in Supplementary Figure S8. The table of detected SIRC hidden copies is presented in Supplementary Data S9. Given that hidden copies are not SIRC in the strict sense of the term, we consider their potential applicability to the study of MITE’s evolution, which is, however, beyond the scope of this work.

### 2.3. SIRC Elements Are Common in MGEs

We investigated the copy numbers of SIRC constituent elements (direct repeats and spacers) and found that spacers have low copy numbers in the ColCEN genome. In contrast, some direct repeat sequences have significant copy numbers across the ColCEN genome (up to 1076 copies), mostly located inside mobile genetic elements. It is common for MGEs to contain sequences similar to those that makeup SIRC DRs—the population of transposons in the DNA/MuDR and RC/Helitron superfamilies contains more than 2000 sequences identical to several types of DRs, and LTR/Gypsy contains up to 10,000 sequences. Particular MGEs that possess over 100 copies of sequences identical to several SIRC DRs are presented in Supplementary Figure S10 (full data on SIRC DRs occurrence in MGEs is in Supplementary Data S5).

**Figure 4:**
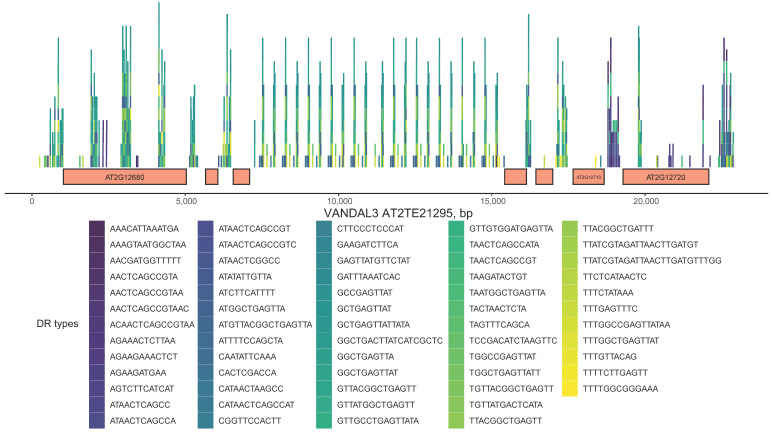
Schematic representation of *VANDAL3* TE AT2TE21295 that contains 270 sequences identical to DRs of 63 types. Colored rectangles above the genes represent sequence positions. DR sequences that overlap are placed above each other.

DRs are often found inside TE tandem repeats. One of the *VANDAL3* members (AT2TE21295, Figure 4) contains 270 sequences similar to DRs of 63 types. Sequences identical to SIRC DRs are found in intergenic spacers, gene parts, terminal sequences, and a large tandem repeat that is located in the middle of this MGE.

We examined which types of SIRC-associated genomic features have SIRC DRs that are most common in MGEs. In general, different MGEs possess regions identical to DR consensuses of MGE-associated SIRC. However, DR consensuses of SIRCs that overlap other genomic features are rare in MGEs.

We conducted SIRC vs. MGE pairwise global-local alignment (Supplementary Figure S11). It was found that MGEs of some families (e.g., *ATHILA2* and *ATHILA6A*) do contain SIRCs similar to SIRC sequences that form clusters (which means that either their SIRC sequences are similar or they possess sequences similar to SIRC but lack SIRC structural features)—which suggests that SIRC propagated and processed to diversification along with MGEs, and emerged inside MGEs when MGEs remain active. On the other hand—nonautonomous *ATREP* MGEs contain very different SIRC sequences, not forming any clusters, suggesting there has been no transposition event of SIRC-containing ATREP after the emergence of SIRC inside them.

### 2.4. SIRC-Associated Genes

SIRCs are found in 356 CDS regions, so-called SIRC-associated genes. The population of protein products of SIRC-associated genes does not differ from the general population of proteins in terms of tissue-specific expression (revealed by PCA of RNAseq data obtained from (https://www.ebi.ac.uk/gxa/experiments/E-CURD-1/Results, accessed on 13 June 2023) (Araport), possible signal peptides and their distribution (targetP), or estimated subcellular localization. PCA on oligopeptide frequencies (1–2, data is not presented) of SIRC-associated proteins shows that there is no difference from the general population of proteins, which suggests that SIRCs in coding sequences do not lead to the emergence of repetitive amino acid patterns. Therefore, we assume that the specific function of SIRCs, if one exists, is carried out in the form of DNA or RNA but not in the form of a protein. 

Considering the fact that SIRCs may be regulatory elements and may influence gene expression by being in any part of a gene, we extended the list of SIRC-associated genes using all in-gene SIRC possible localizations (using the overlap with Araport11 “mRNA” annotation) and obtained a list of 1074 genes. SIRC-associated genes were found in numerous GO categories, among which we underlined 90 genes in reproduction processes, 87 in signaling, 57 in growth, and 16 in immune system processes (Supplementary Figure S12). The only overrepresented GO term found was nuclear transport (14 genes).

### 2.5. Direct Repeat Comparisons

Since SIRC DR consensus sequences vary widely, we conducted comparisons using alignment-free methods. The tetranucleotide frequencies of DR consensuses were calculated and used for principal component and cluster analysis (using Euclidean distances). The PCA showed that all possible SIRC groups are subpopulations of one general population. The repeat similarity between different SIRCs is likely to be unrelated to overlapped genomic features (besides SIRCs from MGEs that apparently were propagated along with MGEs; Supplementary Figure S13).

### 2.6. SIRC-Associated DNA-Binding Proteins

We scanned 2941 full SIRC sequences for cis-regulatory elements (CRE) from PLACEdb and found that 128 SIRCs possess CREs of seven types (Q-values < 0.01). The most abundant were coordinate regulatory elements for antioxidant defense (COREOS, S000469, 133 matches, conserved in the promoter regions of three antioxidant defense genes in rice: cytosolic superoxide dismutase, cytosolic thioredoxin, and glutaredoxin [41]) and “AGTA repeat” of pumpkin (AGTACSAO, S000258, 13 matches, found in the silencer region required for repression of expression of the ascorbate oxidase gene). The full data on CRE occurrence (Q-values < 0.05) in SIRCs is presented in Supplementary Data S14.

Using the data obtained from ReMap db [42], we found that 2255 (of 2941) SIRC have overlaps with TF binding positions.

Using the ReMapEnrich package [43], we estimated the set of DNA-binding proteins strongly associated with SIRCs. Using an FDR of −Log10 (QBH) > 10, we selected five proteins: LDL1 (AT1G62830), SHL (AT4G39100), RVE6 (AT5G52660), TCX6 (AT2G20110), and RVE5 (AT4G01280). The majority of them are associated with histone modifications for transcription repression. LDL1 and LDL2 are thought to control the induction of immunity-related genes [44]. SHL is a histone reader that recognizes H3K27me3 and H3K4me3 and is important for floral repression [45]. RVE5 and RVE6 are transcription factors that play a part in circadian rhythm regulation [46]. TCX6 is a transcriptional repressor of DNA-methylation maintenance genes. TCX6 is a part of the DREAM complex that precludes DNA hypermethylation [47].

### 2.7. SIRC-Associated Histone Modifications

SIRC sites were significantly enriched with several histone modifications: H2A.W (HTA6) and H3K9me2, which are specifically associated with constitutive heterochromatin and transposons of Arabidopsis [48,49]; H3K27me1, a modification also associated with heterochromatin and transposons [50]; and H3.1, a special “replicative” histone variant that is enriched in silent parts of a genome, including regions with H3K27, H3K9, and DNA methylation, densely packed with nucleosomes [51]. The results are consistent with the fact that SIRC is often found in mobile genetic elements and potentially in MITE internal sequences. The full data on SIRC enrichment with histone modification binding sites is presented in Supplementary Data S15.

## 3. Discussion

The highly diverse population of DNA elements constitutes a significant part of the plant genome and contributes to the vast majority of DNA-related processes in a plant cell. In this study, we report the presence of a novel type of repetitive DNA elements in the nuclear genome of an eukaryotic species—*Arabidopsis thaliana*—and we suggest that these new elements may perform yet unknown regulatory functions.

The emergence of SIRC is likely to be related to mobile genetic elements, especially MITE, which is supported by the highly nonrandom way of SIRC distribution in MITEs (that is defined by the highest positional enrichment score of SIRC with MITEs, Figure 3) and the occurrence of a significant part (nearly 50%) of “hidden” SIRC copies in MITEs (mentioned in Section 2.2). We suggest MITEs as the source of SIRCs in the Arabidopsis genome. 

It is common for different MGEs to contain regions identical to SIRC constituent elements—direct repeats (mentioned in Section 2.3); however, this is relevant only for SIRCs that are located within MGEs. We assume that the reason is the MGE recombination process after SIRC was translocated into MGE with MITE. It is also consistent with the peculiarities of SIRC overlap with different genomic features—MGEs and non-MGEs (Supplementary Data S6), as it is common for MITEs to be inserted in different locations, even protein-coding genes [52]. The occurrence of numerous SIRCs in MGEs is probably due to MGE propagation, and the presence of SIRC “hidden” copies in MITEs is probably due to MITE amplification [53]. We assume that not all SIRCs were found to be located in MITEs due to the complexity of MITE detection: it is common to use TIR and TSD for MITE discovery [52], and the loss of these features leads to misdetection of MITEs.

Considering the fact that MITE can possess a distinctive yet relatively simple secondary structure in the single-stranded form [54], we propose that the SIRC may be a special case of MITE with a complex internal structure consisting of small interspaced direct repeats and can potentially be used to study MITE evolution. It is common for MITEs to play a role in gene expression regulation mostly via their inverted repeats [53,54], and SIRCs are found in several inducible protein-coding genes that are associated with interspecies interaction and immunity processes (Supplementary Data S6 and Figure S12). Additionally, the set of DNA-binding proteins strongly associated with SIRC (Supplementary Data S15) is consistent with SIRC being part of the inducible genes of MGEs. These findings led us to speculate that SIRC may be another MITE-derived element that plays a regulatory role regardless of the inverted repeats. 

The emergence of such a complex internal structure as SIRC in MITEs remains unclear. This may be either the consequence of a special yet unknown way of DNA-transposon degradation that leads to MITE formation [52], or the result of tandem repeat evolution—if a MITE possessed a minisatellite with repetitive units conditionally half AT-rich and half GC-rich, the GC-rich half would have a greater mutational rate [55] and would gain substitutions, insertions, and deletions much faster than the AT-rich part. Then the internal structure of MITE would eventually become SIRC, although the probability of this is very low, and it does not explain the emergence of small, inverted repeats inside SIRC direct repeats.

**Figure 5:**
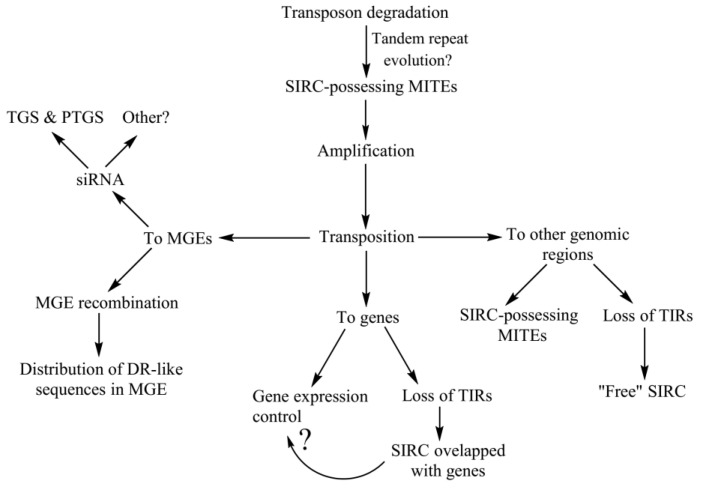
Hypothetical scheme of SIRC emergence, distribution throughout the genome, and possible functions. TIR—terminal inverted repeat, TGS—transcriptional gene silencing, PTGS—post-transcriptional gene silencing, ‘?’—currently unknown.

MGEs are known to be the template for miRNA and siRNA synthesis, pathways that are thought to be evolutionary related and co-opted for immunity-related and regulatory purposes [56]. It is known that there are several proteins participating in both pathways (e.g., AGO4 that carries both hc-siRNA for RdDM and post-transcriptional gene silencing and viRNA for viral DNA silencing [40,57]), and there is some evidence that miRNA and siRNA pathways are colocalized within the nuclear periphery domains—“Dicing Bodies” [58]. We found that SIRCs are present in many siRNA primary transcripts (Figure 2C), which led us to suggest that they are potentially able to play a role in these pathways. The hypothetical scheme of SIRC emergence, distribution, and possible functions is presented in Figure 5.

Therefore, we have reported on novel DNA elements with complex structures in the nuclear genome of a model plant species, *Arabidopsis thaliana*, yet the elucidation of their definite molecular function requires further experimental investigation.

## 4. Materials and Methods

The principal workflow of the current paper is presented in Supplementary Figure S16. 

### 4.1. SIRC Detection and Filtration

First, we conducted putative SIRCs detection using the Col-CEN assembly (GCA_023115395.1: ASM2311539v1) with accurate centromere mapping [59]—since SIRCs are repeated sequences and we expected them to appear among the higher-order repeats of centromeric and pericentromeric regions. The detection was performed using CRISPRCasFinder v4.2.20 software [60], since morphologically, SIRCs resemble clustered regularly interspaced short palindromic repeat (CRISPR) structures in prokaryotes. CRISPRCasFinder utilizes VMatch for the detection of maximal repeats and then merges them together into a possible SIRC cassette. The script used for detection is available at https://gist.github.com/ipetrushin/0e04676ddf3fe0bf2824ff611e787fed (accessed on 13 June 2023). The resulting dataset contained a dozen false positives—tandem repeats, so we conducted a set of filtration procedures: we estimated Trifonov and DUST direct repeat (DR) consensuses DNA complexity using universal motif R package [61] and filtered only DRs with DUST < 0.33 and Trifonov > 0.1, also we applied additional coefficients—DUST*DR length < 11 and Trifonov/DR length > 0.0028. Then we filtered out all DRs with extreme GC-content of 0 or 100%. We extracted spacer sequences of SIRCs, merged them cassette-wise, and filtered SIRCs with a Trifonov complexity of merged spacers greater than 0.15, and Trifonov/spacers number > 0.07. Additionally, we estimated the spacer occurrence in the Col-CEN genome. SIRC must have at least 1 spacer with a copy number in the *A. thaliana* genome less than 100. We performed tandem repeat detection using RepeatMasker v. 4.1.5 software [62] to filter out false SIRCs. Additionally, we conducted tandem repeat detection using complete sequences of SIRCs by the DECIPHER R package [63] with any scores—only the cassettes with no possible tandem repeats were selected. Full sequences of SIRCs were tested for cis-regulatory element (CRE) occurrence (CRE motifs were obtained from the plant cis-regulatory element database PLACEdb [64] at https://www.dna.affrc.go.jp/PLACE/?action=newplace, accessed on 13 June 2023).

We extracted DR consensuses of selected SIRCs and conducted several tests: tetranucleotide frequencies were calculated and used for principal components and cluster analysis (the tree was plotted using ggtree [65]), in-genome copy numbers of DRs were estimated, annotations enriched with DR occurrence were detected, and palindromes within each DR consensus were detected (using Biostrings R package [66]).

### 4.2. Remapping of SIRC—Overlap with Genomic Feature Annotations

The resulted dataset of selected 3050 SIRCs was remapped to reference *A. thaliana* Col-0 genome (TAIR10.1, GCF_000001735.4) using Liftoff software [67], giving a dataset of 2941 SIRCs. We combined TAIR10.1 annotations from Araport11 [68], origins of replication [69], enhancers [70], MGE-derived regulatory inverted repeats [71], small RNAome constructed by Araport11 contributors [68], and miniature inverted-repeat transposable elements (MITE) [72] and calculated overlap of SIRCs with these features. The frequencies of palindrome arm length occurrence among the DRs consensuses of SIRCs overlapped by different annotations were compared using Tukey’s pairwise test. Using the ReMapEnrich package [43], the positional enrichment of SIRCs with a comprehensive set of annotations was calculated. Additionally, we used the data on nonredundant ChIP-Seq peaks from ReMap2022 database [42] to calculate SIRC enrichment with DNA-binding proteins and histone variant binding sites.

### 4.3. Alignment versus MGE Sequences

The sequences of SIRCs that overlapped with MGE annotations were aligned pairwise versus full MGE sequences. The matrix percentages of identities (PID = matched bp/SIRC length × 100%) were used for heatmap construction with hierarchical clustering (hclust(“complete”)) of rows and columns performed with ComplexHeatmap R package [73].

### 4.4. Analysis of SIRC Association with Protein-Coding Genes

The data on SIRC-overlapping protein-coding genes was refined by filtering out SIRC-overlapping introns, and the list of SIRC-associated coding sequences (CDS) was constructed. The products of these CDSs were tested by GO overrepresentation tests and group GO analysis using ClusterProfiler R package [74] and data obtained from org.At.tair.db R package [75]. The mono- and dipeptide frequencies of gene products were used for principal component analysis. The data on protein-protein interactions from Interactome2.0 [76] was used for protein-protein interaction network of SIRC-associated gene products. The expression values of Arabidopsis genes were obtained in the form of FPKM from ExpressionAtlas E-CURD-1 experiments group (at https://www.ebi.ac.uk/gxa/experiments/E-CURD-1/Downloads, accessed on 13 June 2023), provided by Araport team [68], and used for principal components analysis between SIRC-associated and non-SIRC-associated genes.

## Data Availability

The supplementary data files are available with the online version of this paper.

## Supplementary Materials

The following supporting information can be downloaded at: https://www.mdpi.com/article/10.3390/ijms241311116/s1.
