# Supplementary material for: Short Interrupted Repeat Cassette (SIRC)—Novel Type of Repetitive DNA Element Found in Arabidopsis thaliana
Source: Int J Mol Sci. 2023 Jul 5;24(13):11116. doi: 10.3390/ijms241311116 (PMC10342631; doi:10.3390/ijms241311116)

Supplementary Figure S1.

The secondary structure of miRNA ath-MIRf10275 primary transcript. Yellow rectangles are direct repeats. Red line is mature miRNA. Black marks are repeats mismatches (comparing with last repeat). Arcs are base pairs.

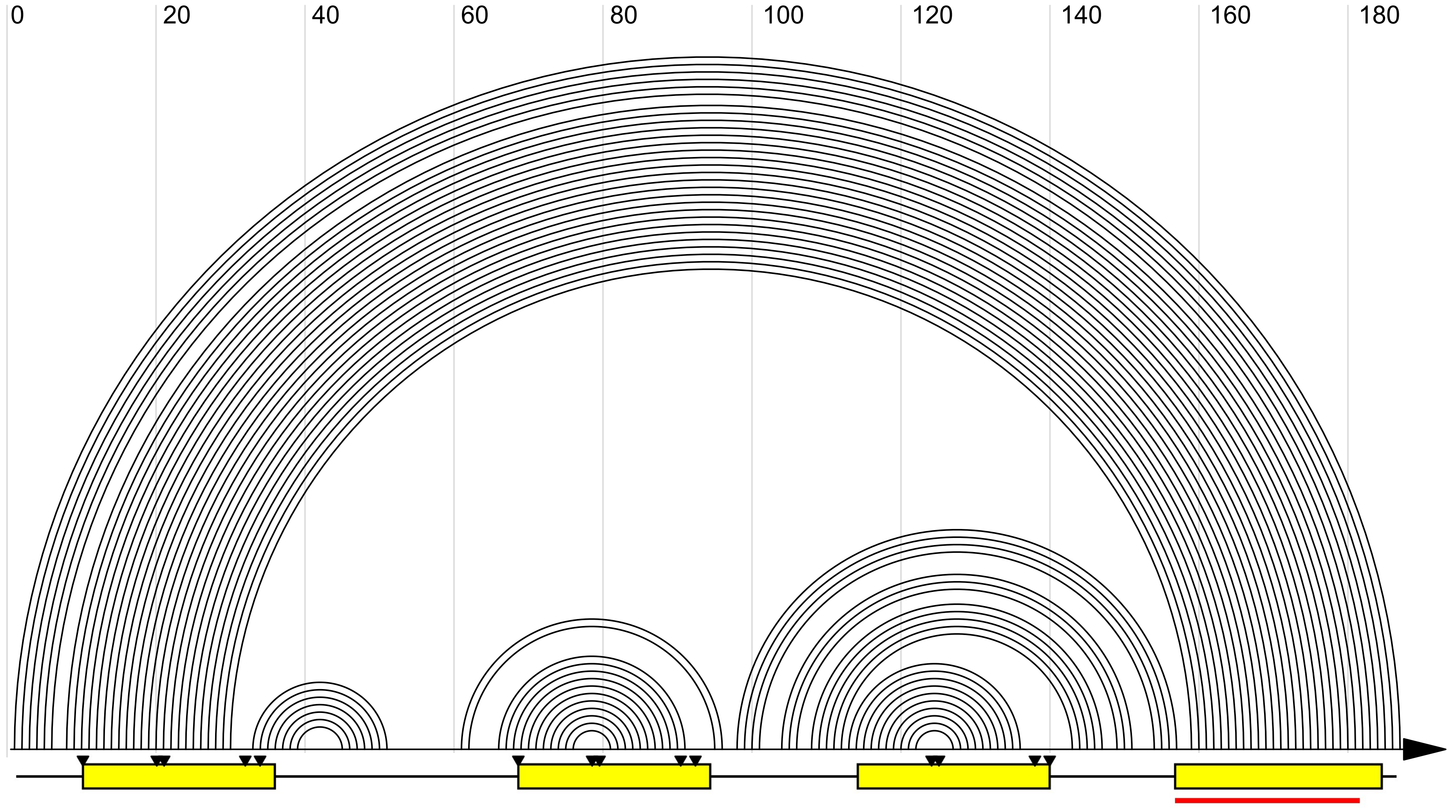

Supplementary Figure S2.

A: Statistics of SIRC DR lengths with spacer numbers; B: GC-content of full SIRC sequences

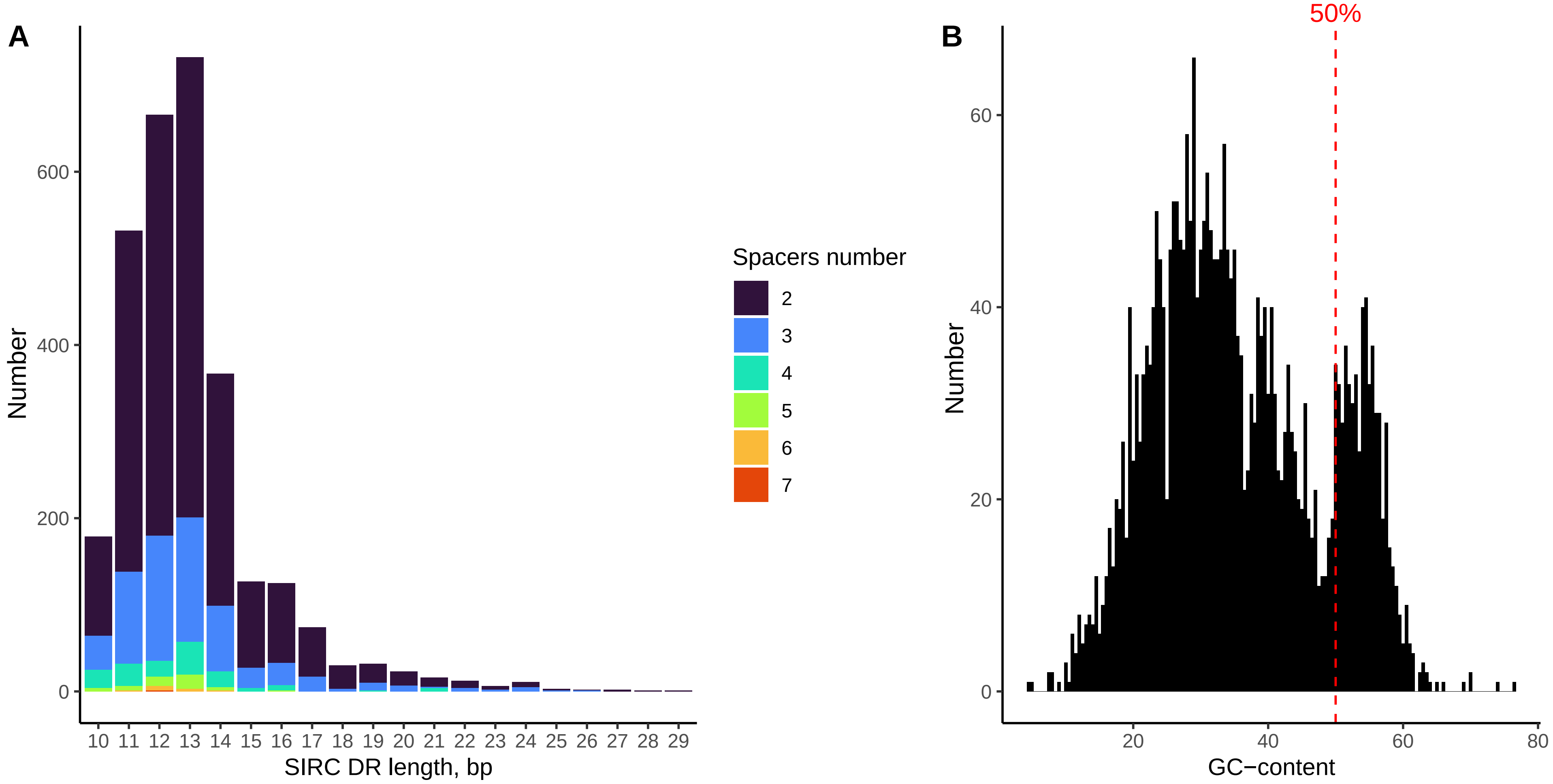

Supplementary Figure S4.

Distribution of SIRCs possessing palindromes in DRs with different arm lengths across Arabidopsis genome (N stands for no palindromes).

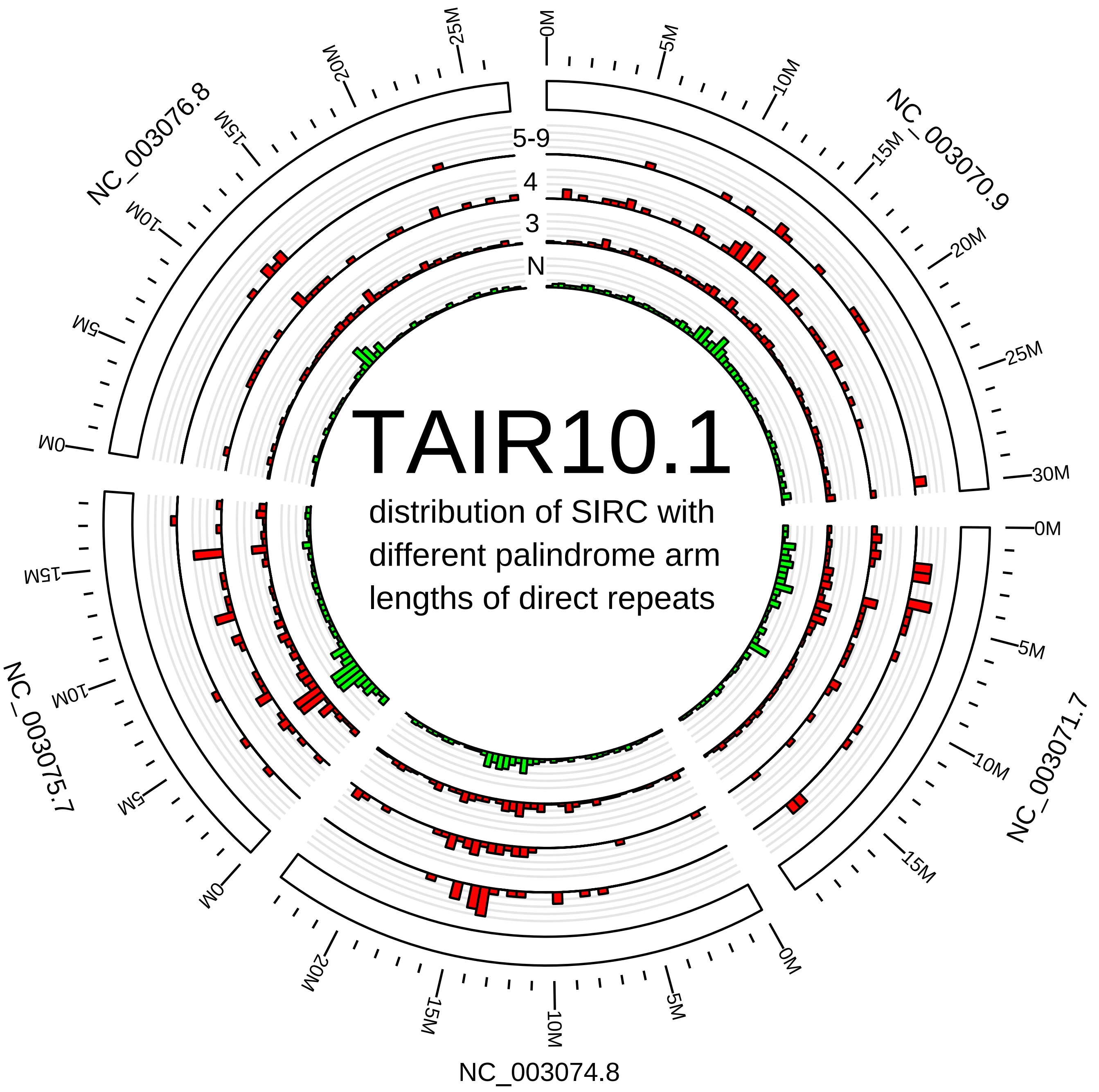

Supplementary Figure S8.
Statistics of Non-MITE mobile genetic containing SIRC hidden copies. The numbers in upper right corner of the frames represents the total number of transposon superfamily members that contain at least 1 hidden copy of SIRC.

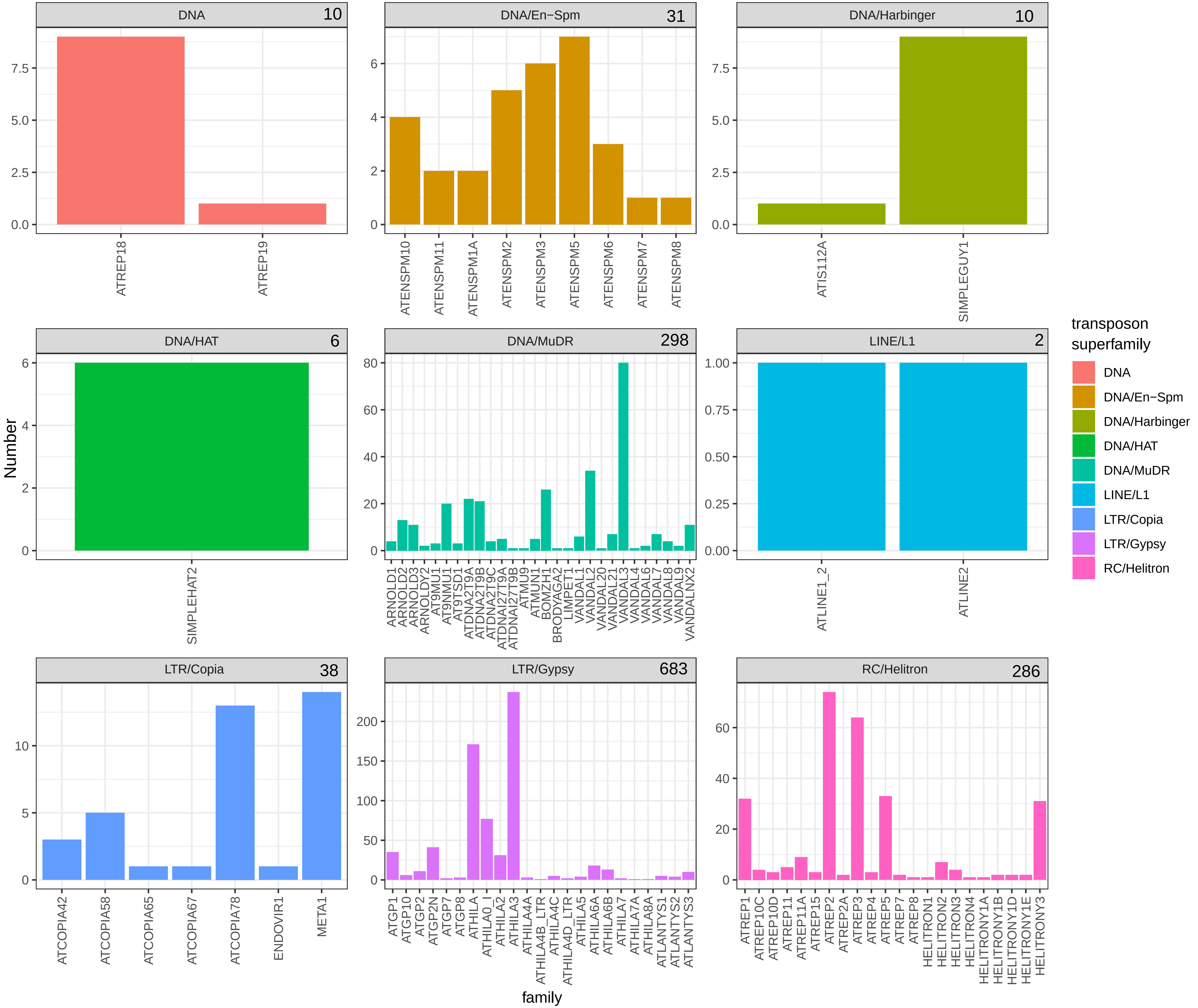

Supplementary Figure S10.

Particular mobile genetic elements that contain 100+ exact copies of different SIRC DRs.

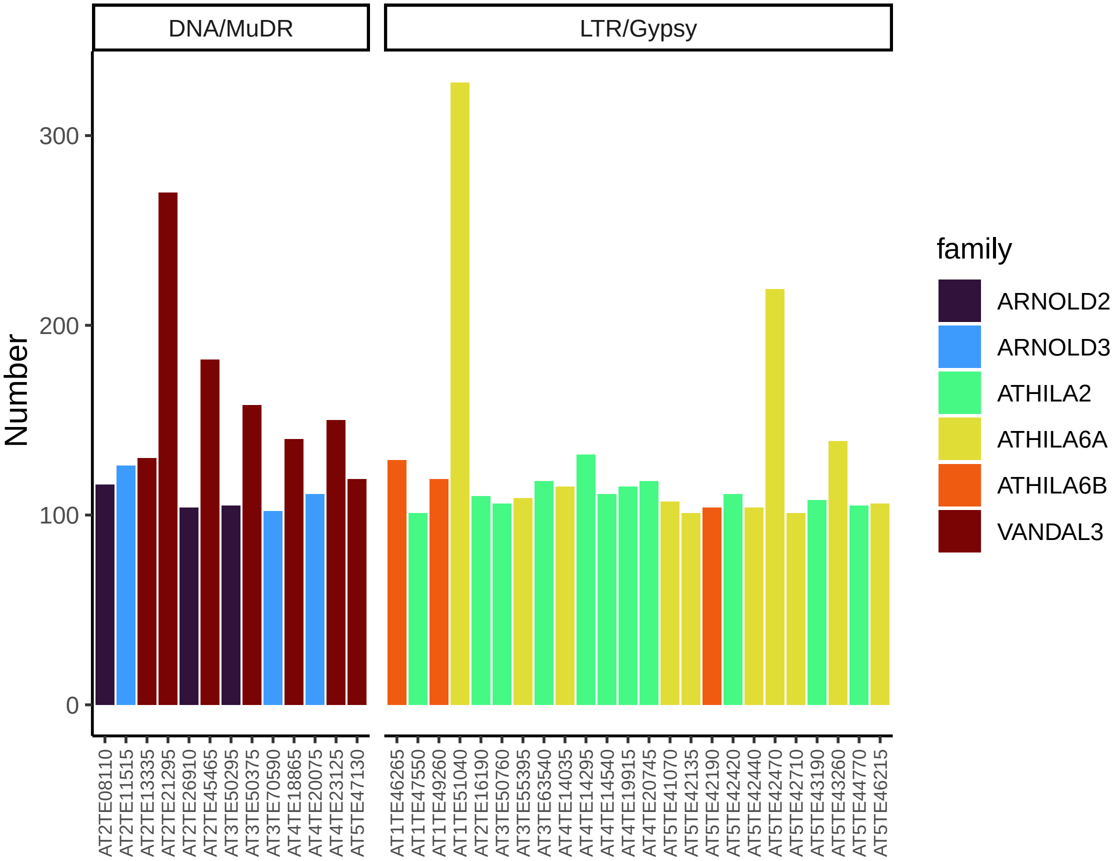

Supplementary Figure S11.

Heatmaps of percentages of identities of global-local pairwise alignments of MGE-located SIRC sequences vs full MGE sequences. A: grouped by MGE superfamilies; B: grouped by MGE families.

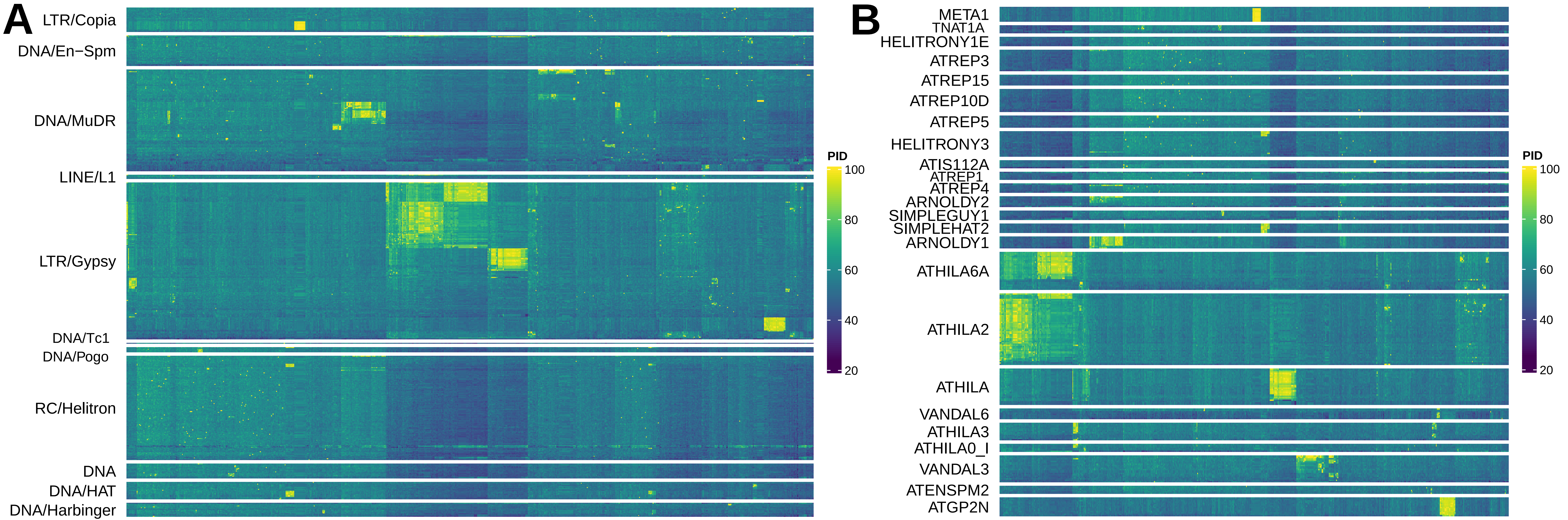

Supplementary Figure S12.
GO group analysis of products of SIRC-associated genes

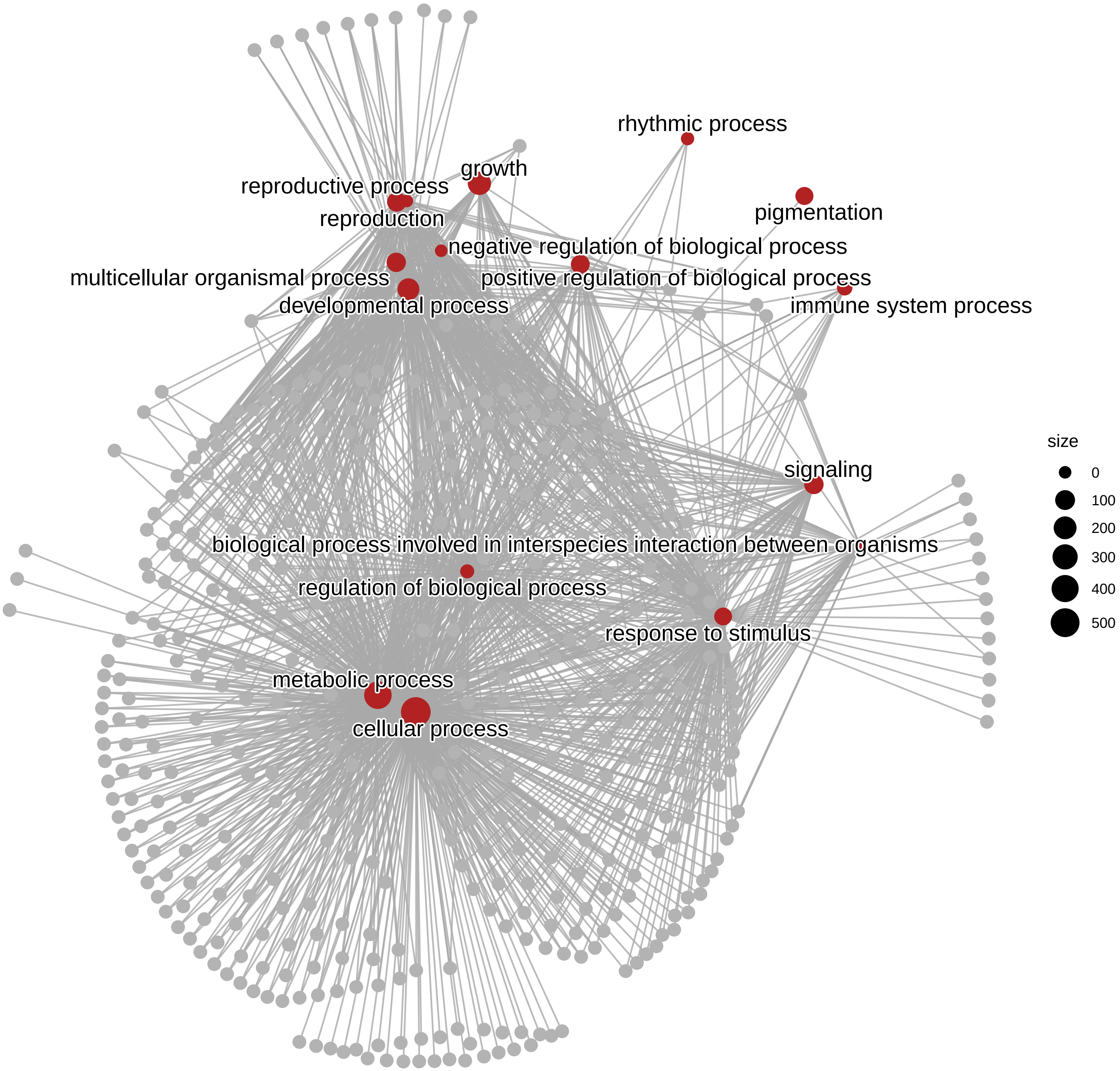

Supplementary Figure S13.

A tree of cluster analysis (method "complete") of tetranucleotide frequencies of direct repeats consensuses of SIRCs, grouped by overlapped annotations.

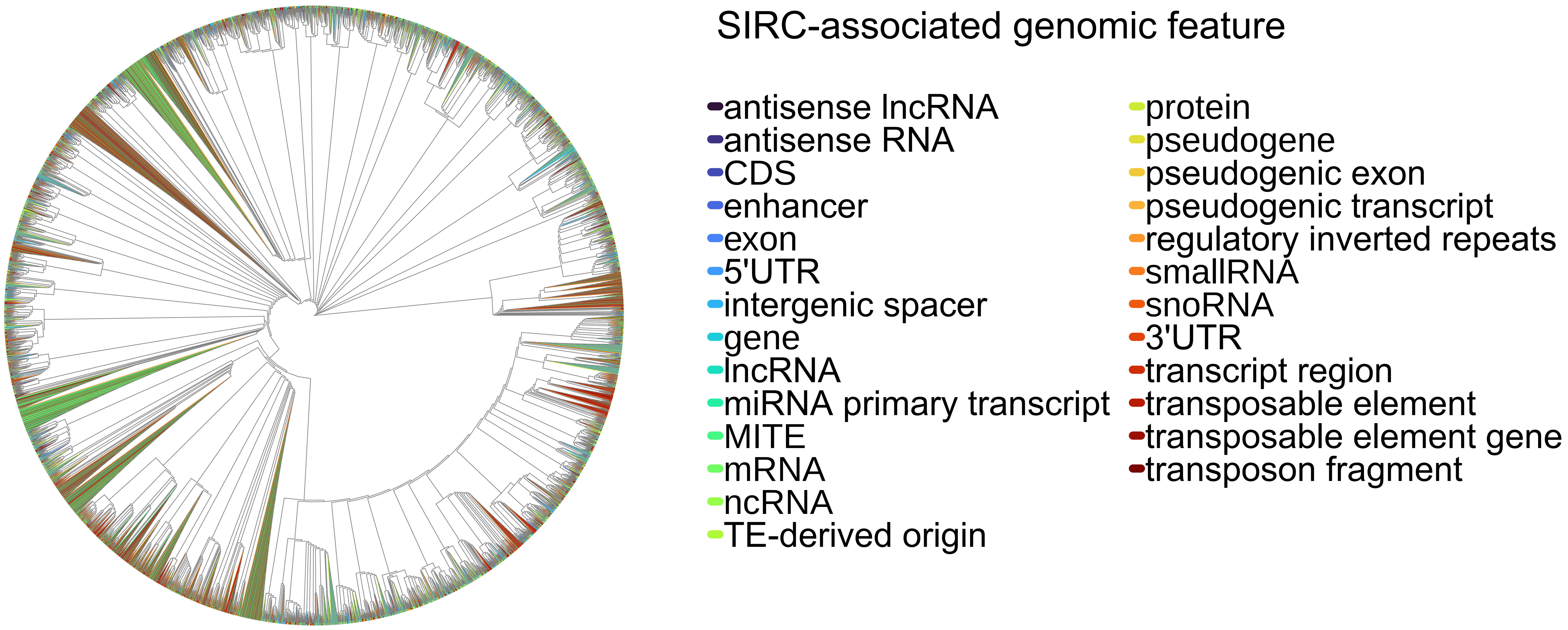

Supplementary Figure S16.
the principal workflow scheme

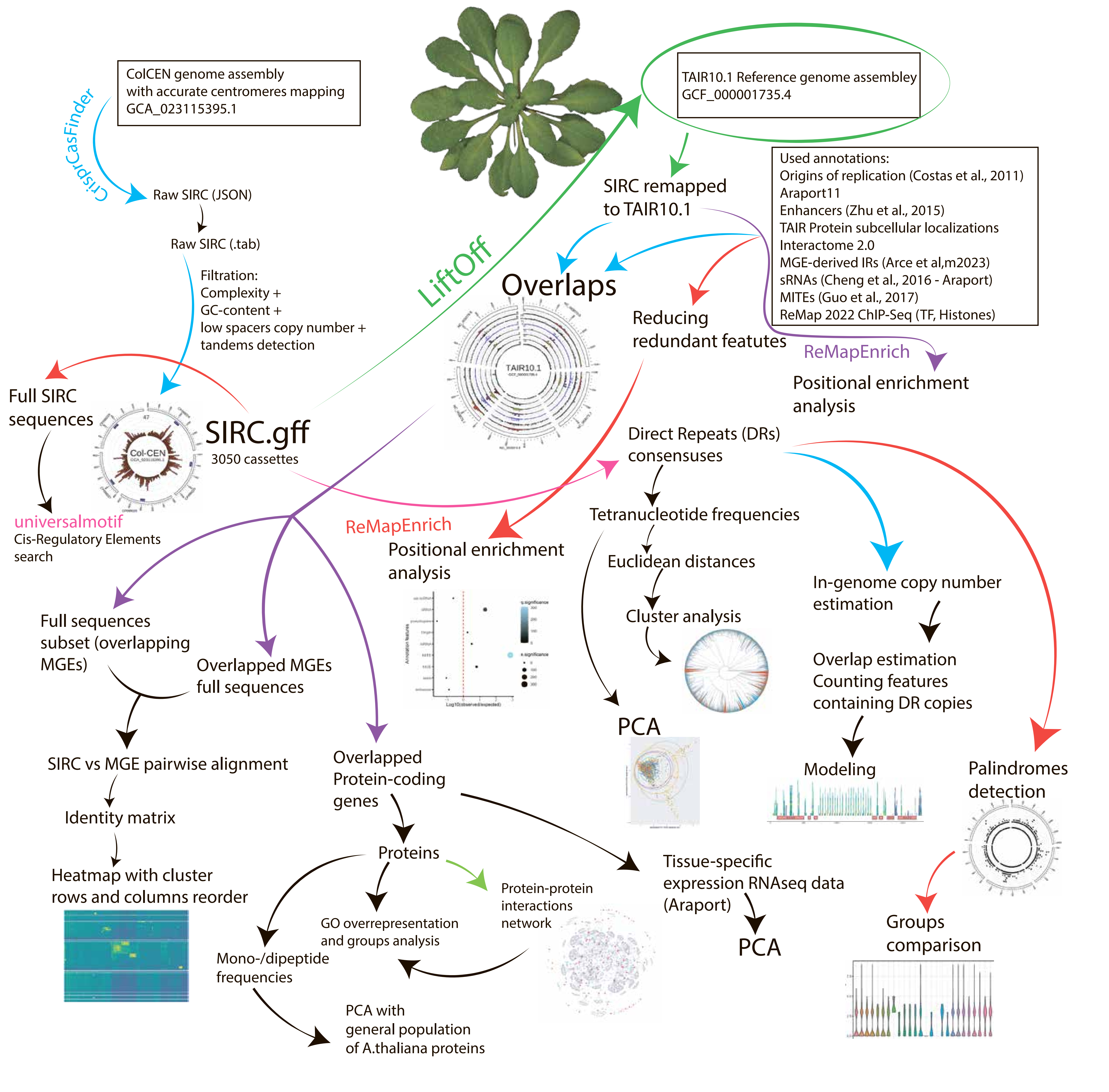

Supplement: Supplementary file 1 [file ijms-24-11116-s001.zip › Supplementary figures.pdf]
